# Response to Letter to the Editor, “Senolytic Treatment Improve Small Intestine Regeneration in Aging”

**DOI:** 10.14336/AD.2024.1262R

**Published:** 2025-12-01

**Authors:** Dong Tan, Chun Xu, Qing Zhu, Gang Fan, Qing-Tian Luo

**Affiliations:** ^1^Department of Gastroenterology, Shenzhen Nanshan People’s Hospital, the 6^th^ affiliated Hospital of Shenzhen University Medical School, Shenzhen, Guangdong, China.; ^2^The Second Affiliated Hospital of the University of South China, Hengyang, Hunan, China.; ^3^Pain Management Department of The Second Affiliated Hospital, School of Medicine, The Chinese University of Hong Kong & Longgang District People's Hospital of Shenzhen, Shenzhen, Guangdong, China.; ^4^Urology department, Shenzhen Nanshan People’s Hospital, the 6^th^ affiliated Hospital of Shenzhen University Medical School, Shenzhen, Guangdong, China

**To the Editor,**

We acknowledge the letter from Dr. Rokutan [1] in response to our paper on the role of dasatinib and quercetin (D+Q) on age-related senescence in the small intestine [2]. Two key points were highlighted by the author for further elucidation or discussion, and we are grateful for the chance to offer further clarification.

In response to the first point concerns regarding the potential acceleration of the beneficial effect of D+Q on old mice during treatment, it is important to consider the characteristics of senescent cells [3]. Senescent cells exhibit morphologic alterations and metabolic reprogramming, and produce a highly active secretome known as the senescence-associated secretory phenotype (SASP) [3], which can lead to local tissue dysfunction and systemic effects [4]. Importantly, D+Q primarily targets senescent cells, leading to their elimination and subsequent anti-aging outcomes, with comparatively minor impacts on young cells [3, 5]. Here, we induced senescence of human normal intestinal epithelial cells through the administration of bleomycin [6]. Senescence-associated beta-galactosidase (SA-β-gal) staining was employed, revealing that the combination of dasatinib and quercetin (D+Q) selectively targeted SA-beta-gal positive cells. Interestingly, D+Q treatment did not exhibit efficacy in cells that had not been exposed to Zeocin (a member of the Bleomycin family) (Fig. 1. A-C). Our research (also see [2]) indicated that the removal of senescent cells by D+Q potentially improved the endo-environment of intestinal cells. However, it is important to note that since D+Q primarily targets senescent cells and the process of senescence in our study model represents natural aging, the action of D+Q may not result in a positively spiraling effect, despite the possibility of increased D+Q uptake.

The second point regards the heterogeneity of p16 expression in cellular senescence. P16 and p21 are commonly used markers for senescent cells and have been widely employed in identifying senescent cells in various tissues under aging or pathological conditions [7]. Our previous study demonstrated that aged small intestinal epithelium samples exhibited increased expression of p21 and p16 proteins, which were effectively suppressed by D+Q treatment in 16-month-old mice [2]. Furthermore, immunofluorescence staining revealed an upregulation of p16 expression in small intestinal villi epithelial cells of old mice compared to young mice, which was subsequently rescued by D+Q treatment (Fig. 1. D-E). These result is consistent with the study in aging mice that D+Q-treated aged mice exhibit a pronounced reduction in senescent cell presenting by diminished levels of p16 and p21 expression within both the small and large intestine, when compared to the control mice [8]. While D+Q treatment has shown significant reduction in senescent cell presence indicated by decreased levels of p16 and p21 expression in aged mice, individual variability in response to the treatment cannot be ruled out.

It has been noted that p16 may not be a specific orsensitive marker for cellular senescence, as not all cells highly expressing p16 are necessarily senescent [9], and some senescent cells do not express p16 [10, 11]. Nevertheless, the Mouse Ageing Cell Atlas, encompassing sequencing data from 23 mouse tissues, provided insights into age-related changes in senescent cells in older mouse cohorts, revealing a significant upregulation of p16 expression with advancing age [12]. In conjunction with existing literature, our findings suggest that p16 may serve as a potential indicator of cellular senescence specifically in the small intestinal epithelium.

**Figure 1.**
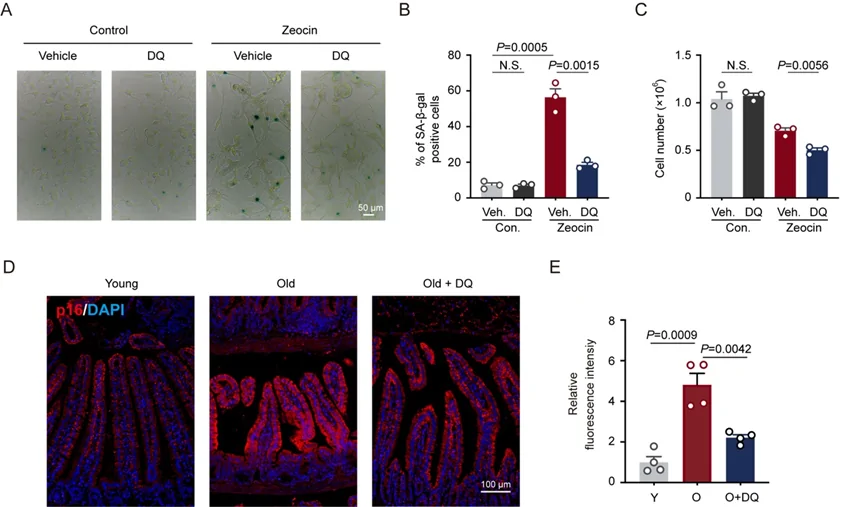
**D+Q removes senescent cells and rescues p16 expression in aged small intestine**. (**A-C**) β-galactosidase activity staining showing the senescent cells in NCM460 cells induced with or without Zeocin followed by treated with or without D+Q. (**A-B**) Quantification data of percentage of SA-β-gal positive cells and cell numbers in part (C). The experiment was repeated three times. Scale bar, 50-μm. (**D-E**) Immunofluorescence staining showing an up-regulation of p16 expression in jejunal villi epithelial cells in old mice, which were rescued by D+Q treatment. (**D**) Semi-quantification of expression levels of p16 present in part (E) was performed using ImageJ software. *n*=4 mice in each group. Red, p16; blue, DAPI. Scale bar, 100-μm. Data are expressed as mean ± SEM. *P* values were determined using a one-way ANOVA followed by a Tukey’s post hoc test (Figure. 1B-C, E). Con. control; Veh., vehicle; Y, young; O, Old; DQ, dasatinib and quercetin; N.S., no significance.

## Methods

Mouse models: Young (3-month-old)-, Old (vehicle-treated) (16-month-old)-, D+Q treated old (16-month-old) male C57BL/6 mice were used. Mice were purchased from Shenzhen TopBiotech Co., Ltd and maintained at the breeding facility of the Animal Center of Shenzhen Nanshan People’s Hospital in individually ventilated cages under standardized conditions that included a 12-hour dark-light cycle and free access to standard chow, and water *ad libitum*. Animal experiments were carried out in accordance with recommendations in the National Research Council Guide for Care and Use of Laboratory Animals and in compliance with relevant ethical regulation for animal testing and research, with the protocols approved by the Institution Animal Care and Use committee of Shenzhen Nanshan People’s Hospital. There are no ethical concerns.

Drug administration: Mice were administered a senolytic cocktail containing 5 mg/kg dasatinib (Selleck Chemicals, S1021) and 50 mg/kg quercetin (Sigma-Aldrich, Q4951) as previously described [2]. Briefly, Dasatinib and quercetin were dissolved in 10% polyethylene glycol 400 (PEG 400; Sigma-Aldrich, 25322-68-3). Mice were gavaged bi-weekly for 4 months with D+Q or vehicle (10% PEG 400).

Immunofluorescence staining: Small intestines were harvested immediately after killing and washed with PBS. The jejunum was opened longitudinally and coiled with the mucosal layer inward using a wooden stick like Swiss Rolls and then fixed in 4% paraformaldehyde. Tissues were dehydrated, cleared and embedded in paraffin, cut into serial 5-μm-thick sections for immunofluorescence staining. The tissue sample sections were permeabilized using 0.1% Triton X-100 (Sigma-Aldrich) and blocked with 5% goat serum in PBS, and incubated overnight with rabbit polyclonal anti-p16 antibodies (10883-1-AP, Proteintech, 1:100) at 4 °C. The next day, sections were washed several times with PBS, followed by incubation with species-specific secondary antibodies conjugated to 647 nm fluorophores which were raised in goat (1:1000), stained in a humidified chamber at 37 °C for 1 h. Finally, the samples were washed with PBS and counterstained with 4,6-diamidino-2-phenylindole (DAPI). Stained sections were examined using an FV3000 confocal laser scanning microscope system (Olympus). Images were taken using the same acquisition settings. For quantification analysis, at least three sections from each mouse were selected, and at least three mice were analyzed in each group. ImageJ (National Institutes of Health, USA) was used to calculate fluorescence intensity.

NCM460 cell culture and treatment: The NCM460 cells used in this experiment were acquired from INCELL (San Antonio, TX, USA). The cells were cultured in a RPMI-1640 medium (Gibco) with 10% fetal bovine serum, penicillin (100 U/ml), and streptomycin (100 μg/ml) in a humidified at 37°C in a humidified 5% CO_2_ incubator. NCM460 cells (1 × 10^5^) cultured above were seeded onto 6-well plates. After culturing for 24 h, NCM460 cells were treated with or without Zeocin (10 μM, Beyotime, ST1451) for 48 h to induce cellular senescence, and followed by treated with Dasatinib (0.2 µM) + Quercetin (20 µM) for 24 hours.

Senescence associated ß-galactosidase activity staining: Senescence associated β-galactosidase (SA-β-gal) activity was detected in NCM460 cells by using a β-Galactosidase Staining Kit (Beyotime, C0602) according to manufacturer's protocol. Briefly, cells were fixed and stained with β-Galactosidase Staining Solution (pH of 6.0) overnight in a dry incubator at 37°C. Brightfield and phase (same field) were scanned with EVOS® FL Auto Imaging System at 10X objective. SA-β gal positive (blue) cell numbers were counted on brightfield pictures. Total cell numbers were counted on corresponding phase pictures. Senescence staining was quantified as the percentage of SA-β-gal positive cell number over total cell number. For each individual experiment, at least 400 total cells were counted for each group.

Statistical analysis: All data are reported as the mean ± SEM as indicated in the figure legends. In most cases, each data point corresponds to an individual animal. All experimental data shown has been reliably reproduced by multiple lab members. All biochemical data were statistically analyzed with GraphPad Prism 9.0 software. Whether the data were normally distributed was tested using the Shapiro-Wilk normality test. A one-way ANOVA followed by a Tukey’s post hoc test was used to determine the statistical differences. If the data were not normally distributed, comparisons were performed by non-parametric test. *P*-values < 0.05 were considered statistically significant.
